# Could nutrition status predict fatigue one week before in patients with nasopharynx cancer undergoing radiotherapy?

**DOI:** 10.1002/cam4.7191

**Published:** 2024-04-25

**Authors:** Jihong Song, Xinru Yang, Jieling Wu, Zilan Wu, Litao Zhuo, Jinsheng Hong, Li Su, Wenlong Lyu, Jinru Ye, Yan Fang, Zhiying Zhan, Hairong Zhang, Xiaomei Li

**Affiliations:** ^1^ School of Nursing Health Science Center, Xi'an Jiaotong University Xi'an China; ^2^ School of Nursing Fujian Medical University Fuzhou China; ^3^ Department of Radiotherapy Cancer Center, the First Affiliated Hospital of Fujian Medical University Fuzhou China; ^4^ Department of Radiotherapy National Regional Medical Center, Binhai Campus of the First Affiliated Hospital, Fujian Medical University Fuzhou China; ^5^ Key Laboratory of Radiation Biology of Fujian Higher Education Institutions, The First Affiliated Hospital, Fujian Medical University Fuzhou China; ^6^ Nursing Department The First Affiliated Hospital of Fujian Medical University Fuzhou China; ^7^ Department of Epidemiology and Health Statistics Fujian Provincial Key Laboratory of Environment Factors and Cancer, School of Public Health, Fujian Medical University Fuzhou China; ^8^ Fujian Center for Disease Control and Prevention Fuzhou China

**Keywords:** body composition, fatigue, intensity‐modulated, longitudinal studies, nasopharyngeal carcinoma, radiotherapy

## Abstract

**Background:**

Patients with nasopharyngeal carcinoma (NPC) undergoing radiotherapy experience significant fatigue, which is frequently underestimated due to the lack of objective indicators for its evaluation. This study aimed to explore the longitudinal association between fatigue and nutrition status 1 week in advance.

**Methods:**

From January 2021 to June 2022, a total of 105 NPC patients who received intensity‐modulated radiation therapy were enrolled in the observational longitudinal study. The significant outcomes, including the Piper Fatigue Scale‐12 (PFS‐12), the Scored Patient‐Generated Subjective Global Assessment (PG‐SGA), four body composition indices, and the Hospital Anxiety and Depression Scale (HADS), were assessed weekly from pre‐treatment until the completion of radiotherapy (T0–T7) to explore their relationship.

**Results:**

The trajectories of PFS‐12 and all dimensions for 105 participants reached a peak during the fifth week. Sensory fatigue consistently received the highest scores (T0 = 1.60 ± 2.20, T5 = 6.15 ± 1.57), whereas behavior fatigue exhibited the fastest increase over time (T0 = 1.11 ± 1.86, T5 = 5.47 ± 1.70). Higher PG‐SGA scores were found to be weakly explainable for aggravating fatigue (*β* = 0.02 ~ 0.04). Unlike generalized additive mixed models, marginal structural models (MSM) produced larger effect values (*β* = 0.12 ~ 0.21). Additionally, body composition indices showed weakly negative relationships with fatigue in MSMs one week in advance.

**Conclusions:**

The PG‐SGA may be a more accurate predictor of future‐week fatigue than individual body composition indicators, particularly when HADS is controlled for as a time‐dependent confounder.

## 1 INTRODUCTION

In southern China, the incidence rate of nasopharyngeal carcinoma (NPC) is nearly twice compared with the global incidence.^1^ Depending on the specific anatomical sites and high radiotherapy sensitivity of NPC, patients undergo 6–7 weeks of intensity‐modulated radiation therapy alone or with concurrent chemotherapy (CCRT), resulting in various adverse effects and discomfort. Cancer‐related fatigue (CRF) and malnutrition are common systemic adverse reactions found to increase significantly in patients with NPC during CCRT,^2^, ^3^ with 22.2% of patients reporting severe fatigue at the end of CCRT^4^ and 53.1% having suspected malnutrition after diagnosis.^5^ It has been demonstrated that early and effective identification reduces fatigue and improves quality of life.^6^

Cancer‐related fatigue (CRF) is considered a subjective experience that is currently assessed by self‐reported scales and is often under‐reported due to barriers from patients/caregivers/clinicians. For instance, CRF is believed to be a natural treatment‐related reaction for which there are no effective interventions, and the report is meaningless, which could alter or halt treatments.^6^ Hence, developing some objective measurement indicators is necessary to improve intervention efficiency.

Body composition indexes have received increasing attention over the last decade as important indicators of nutritional status. Some were identified as significant prognostic factors for toxicity treatment^7^ and cancer survival.^8^, ^9^, ^10^, ^11^ However, the association between fatigue and body composition is uncertain. The existing studies are mainly cross‐sectional studies, and the body composition indexes selected by each study are different.^12^ A study on 104 colorectal cancer patients showed that visceral fat mass, intramuscular fat mass, and skeletal muscle index were not associated with fatigue.^13^ Other three cross‐sectional studies^14^, ^15^, ^16^ concluded that the reduced trunk skeletal muscle mass^14^ and skeletal muscle index^15^, ^16^ could predict the intensity of fatigue in only males. However, Aleixo and colleagues found in a cross‐sectional analysis of 99 patients with early‐stage breast cancer that those with a low BMI and skeletal muscle index reported less fatigue during chemotherapy.^14^ Furthermore, a cross‐sectional investigation and Spearman correlation analysis were adopted in the above studies, which could not reveal the relationship between the dynamic trend of fatigue and nutritional status.

Few longitudinal studies have examined the relationship between baseline body composition and the trajectory of fatigue. Van Baar investigated the relationship between baseline skeletal muscle/fat‐related indices and fatigue in 646 patients with colon cancer during the first 2 years after diagnosis. The results showed that patients with high subcutaneous fat had stronger fatigue at diagnosis, while those with low skeletal muscle radiation density had stronger fatigue 6 months after diagnosis.^15^ Another secondary analysis of a longitudinal, nationwide study of 565 breast cancer patients revealed that post‐chemotherapy fatigue scores remained substantially higher for obese patients than normal‐weight patients.^16^ The unstable conclusions indicated the baseline body composition could not predict the trajectory of fatigue.

Previous investigations on patients with NPC showed that the trajectories of nutritional status and fatigue were in the same direction but nonsynchronous.^17^, ^18^, ^19^ As a result, it can be deduced that there may be a time window issue regarding nutritional status alterations and fatigue, which has been the subject of few studies. The present study focused on adults with NPC who underwent radiotherapy alone/with chemotherapy and aimed at the following issues: (1) To explore the trajectories of CRF, nutrition status, and body composition using an observational longitudinal study; (2) To identify the factors that contribute to multiple‐dimension fatigue; (3) To reveal if nutrition status, body composition 1 week in advance could predict fatigue and what about the independent strength.

## 2 PARTICIPANTS AND METHODS

### 2.1 Participants and treatment regimen

An observational, longitudinal study was designed for this research. Patients were enrolled from January 2021 to June 2022 in the Department of Radiation Oncology, the First Affiliated Hospital of Fujian Medical University. The patients were included if they met the following criteria: (1) NPC were diagnosed for the first time by histopathological examination (Nasopharyngeal carcinoma 2018 edition 8th UICC/AJCC staging criteria); (2) Age ≥ 18 years old and knew their diagnosis; (3) Received intensity‐modulated radiation therapy alone/with chemotherapy therapy. The subjects were excluded for those who (1) were diagnosed with cognitive or mental disorders or physical dysfunction or receiving antidepressants or psychotherapy, and (2) quit radiotherapy halfway. The prescribed doses of nasopharynx gross tumor volume, lymph node gross tumor volume, clinical target volume 1, and clinical target volume 2 were 66–76 Gy, 66–70 Gy, 60–62 Gy, and 50–56 Gy/30–33 Fx, respectively for a total of 6–7 weeks. The nutrition status of all the patients was assessed by the Scored Patient‐Generated Subjective Global Assessment (PG‐SGA) weekly, and nutritional therapy was performed according to European Society for Clinical Nutrition and Metabolism (ESPEN) practical guideline.^20^

### 2.2 Assessments of outcomes

The Piper Fatigue Scale‐12 (PFS‐12) was used to assess fatigue for participants.^21^ There are 12 items, with four subscales measuring behavioral, affective, sensory, and cognitive/mood dimensions (three items in each subscale). The behavioral subscale pertains to the influence of tasks performed in everyday life, whereas the affective subscale has items associated with the emotional aspects of fatigue. The sensory subscale is concerned with the physical symptoms of fatigue, and the cognitive/mood subscale assesses an individual's mental and mood status. Each item is rated on an 11‐point (0–10) metric scale, where higher scores indicate a higher level of fatigue, with the highest score being 10. The score of each subscale is the average score of the included items. The PFS‐12 has been verified to be an effective tool for assessing CRF in patients who are in active treatment or immediate posttreatment. Cronbach's alpha value of the PFS‐12 in our participants was 0.98; the reliability ranged from 0.60 to 0.95, and the test–retest reliability was above 0.86.^22^

The nutritional status of subjects was assessed using the Scored Patient‐Generated Subjective Global Assessment (PG‐SGA),^23^, ^24^ combined with patients' self‐reports and physicians' assessments. The following factors were considered: (1) Box A: Patient perception and patient‐reported concerns; (2) Box B: Variables of risk for malnutrition or prediction of the degree of nutritional deficit; (3) Box C: Intervention options for nutritional intake and nutrition impact symptoms to prevent or reverse malnutrition and weight loss; (4) Box D refers to known prognostic variables. Except for Box A, the supervising doctors assessed the other three Boxes. A meta‐analysis showed the pooled sensitivity for PG‐SGA was 0.964 and its specificity was 0.905; this value was recommended as the best diagnostic performance in patients with cancer.^25^ In this study, only the total scores of PG‐SGA were analyzed.

In this analysis, body composition indexes covered weight, body mass index, body fat rate, and lean body weight. Based on bioelectricity impedance, patients were assessed in light clothing barefoot (no shoes and socks) on a smart body fat scale (Yolanda mini+, Huawei Hilink Ltd., China). Bioelectricity impedance analysis measured the electrical impedances when a weak alternating current passed through different body compositions. Bioelectricity impedance analysis is widely adopted due to the advantages of the noninvasive, convenient operation, and rich information.

Hospital Anxiety and Depression Scale (HADS),^26^ composed of 14 items and two subscales, was used to examine the psychiatric symptoms of patients during treatment. Each item is rated from 0 (no sense) to 3 (severest) points, with higher scores mean severe anxiety and depression. Cronbach's alpha of the Chinese version of HADS was ≥0.84 in cancer patients, construct validity was 0.83, and concurrent validity was 0.40 ~ 0.55.^27^ The total scores were analyzed in the study.

The 10‐item Resilience Scale (RS) revised by Campbell‐Sills^28^ was adopted to assess self‐recovery from stress‐related events, with content validity between 0.83 and 1, Cronbach's alpha value of 0.877 ~ 0.879, and test–retest reliability of 0.73 ~ 0.945.^29^, ^30^ The social support status of patients was measured using the Social Support Rating Scale (SSRS),^31^ with a test–retest reliability of 0.92 and internal consistency reliability of 0.94. The physical activities of patients were divided into three levels (from low to high) using the Chinese version of the International Physical Activity Questionnaire (IPAQ),^32^, ^33^ with test–retest reliability ranging from 0.78 to 0.89 and intra‐class correlation coefficients ranging from 0.74 to 0.95.^33^ The correlation between PA and the criterion accelerometer was unideal (*r* = 0.35), as reported by other widely used self‐reported PA questionnaires used for PA surveillance.^34^

### 2.3 Data collection

All eligible subjects were approached by trained research group members and asked to complete the surveys (demographic characteristics and all the scales) before radiotherapy (T0). Otherwise, the PFS‐12, PG‐SGA, HADS, and body composition were assessed weekly in person until the end of radiotherapy (T1–T7). Demographic characteristics, including age, sex, education status, marital status, settlement, family income (household per capita income/month), smoking, and drinking history, were collected in the first survey. Clinical factors such as tumor stage, induced chemotherapy, and CCRT cycle were extracted from medical records. Two researchers reviewed data for missing items immediately after the interview to reduce errors.

### 2.4 Statistical analysis

The software EmpowerStats 4.1 (X&Y Solutions, Inc., BSN, MA, USA) was designed in R language to analyze the data. Statistical significance was defined as a two‐sided *p*‐value less than 0.05.

Means, standard deviations, and 95% credibility intervals were used to describe continuous variables (e.g., age, the scores of the PFS‐12, HADS, RS, SSRS, PG‐SGA, and body composition). Numbers and percentages were adopted to count variables (e.g., sex, smoking, drinking, tumor stage, levels of physical activities).

Univariate regression analysis was adopted to disclose the single effect of demographic (sex, age, marital status, education, settlement, incoming), clinical (UICC/AJCC stage of NPC, induced chemotherapy cycle, and CCRT cycle, total radiation dose, fractionated dose), baseline lifestyle (smoking, drinking, physical activity), psychiatric (baseline resilience, hospital anxiety, and depression), and social support characteristics on fatigue.

A generalized additive mixed model (GAMM), a combination of the generalized additive model and mixed model, was easily accommodated in exponential distribution longitudinal data and was used to reveal the changes in fatigue, nutrition, and body composition over time. Subsequently, we investigated the longitudinal relationships between the dependent variable of fatigue (Y) and the independent variables of nutrition and body composition as of 1 week ago (X). The aforementioned demographic data, clinical, baseline lifestyle, psychiatric, and social support characteristics were regarded as potential confounders and included as covariables in the models. Three GAMM models were conducted during the study to assess the stability of results: (1) Null model: the absence of covariables. (2) Model I: the above covariables were screened out by changing the X coefficient (β) by a threshold greater than 10%^35^, ^36^ while adding the covariate to the basic model or removing it from the full model, provided that a collinearity diagnosis was confirmed (coefficient of variance expansion <10)^36^; (3) Model II: meeting the Model I or the covariate *p* < 0.1 in the univariate model. The above models included the intercept and time as random terms and the other variables as fixed effects. The interaction terms between body composition a week ago and the examination time have been evaluated. In accordance with the likelihood‐ratio test, time was modeled as a continuous variable in this investigation.

The marginal structural model (MSM) was conducted to control the effect of HADS, considered a time‐dependent confounding factor, and was synchronously collected with fatigue, nutrition, and body composition in this study. The analysis in MSM used the HADS total score obtained concurrently with fatigue; other adjusted confounders were the same as in GAMM Model I.

The study protocol was approved by the Institutional Review Board (IRB) of Fujian Medical University (FMU2021[114]). Each participant provided informed content before each survey.

## 3 RESULTS

### 3.1 The demographic and clinical characteristics of participants

Between January 2021 and June 2022, 105 patients completed the initial assessment, and 98 subjects finished the entire 8‐point assessment in the longitudinal study **(**Figure S1 **)**. Seven subjects lost follow‐up; four of them withdrew for personal reasons; and three of them quit radiotherapy for a combination of multiples seasons. And no patient interrupt radiotherapy single reason of fatigue or malnutrition. All the data were included in the analysis. Table 1 presents the main demographic and clinical characteristics of the participants.

**TABLE 1:** Demographic and clinical characteristics of participants (*n* = 105).

Variables	Value
Age (years, Mean ± SD)	47.30 ± 11.63
Sex (*N*, %)	
Male	81 (77.14%)
Female	24 (22.86%)
Marital status (*N*, %)	
Never married or Divorced	17 (16.19%)
Married	88 (83.81%)
Education (*N*, %)	
Junior high and below	57 (54.29%)
Senior high and above	48 (45.71%)
Settlement (*N*, %)	
Country	39 (37.14%)
Town	33 (31.43%)
City	33 (31.43%)
Family incoming (*N*, %)	
≤5000 Y/month	41 (39.05%)
>5000 Y/month	64 (60.95%)
Smoking (*N*, %)	
Yes or ever	50 (47.62%)
No	55 (52.38%)
Drinking (*N*, %)	
Yes or ever	44 (41.90%)
No	61 (58.10%)
UICC/AJCC stage (*N*, %)	
II and below	16 (15.24%)
III and above	89 (84.76%)
Total radiation dose (*N*, %)	
﹤70 Gy	61 (58.65%)
≥70 Gy	43 (41.35%)
Fractionated dose (*N*, %)	
﹤2.15 Gy	70 (67.31%)
≥2.15 Gy	34 (32.69%)
Induced chemotherapy cycle (*N*, %)	
≤2 times	31 (29.52%)
≥3 times	74 (70.48%)
CCRT cycle (*N*, %)	
≤1 times	43 (40.95%)
≥2 times	62 (59.05%)
Physical activity level (*N*, %)	
Low	10 (9.62%)
Medium	31 (29.81%)
High	63 (60.58%)
Resilience Scale (Mean ± SD)	60.89 ± 20.03
Social Support Rating Scale (Mean ± SD)	41.62 ± 7.30

Abbreviation: CCRT, concurrent chemoradiotherapy.

### 3.2 The dynamic variation of the PFS‐12, the PG‐SGA, and body composition over time during treatment

**FIGURE 1:**
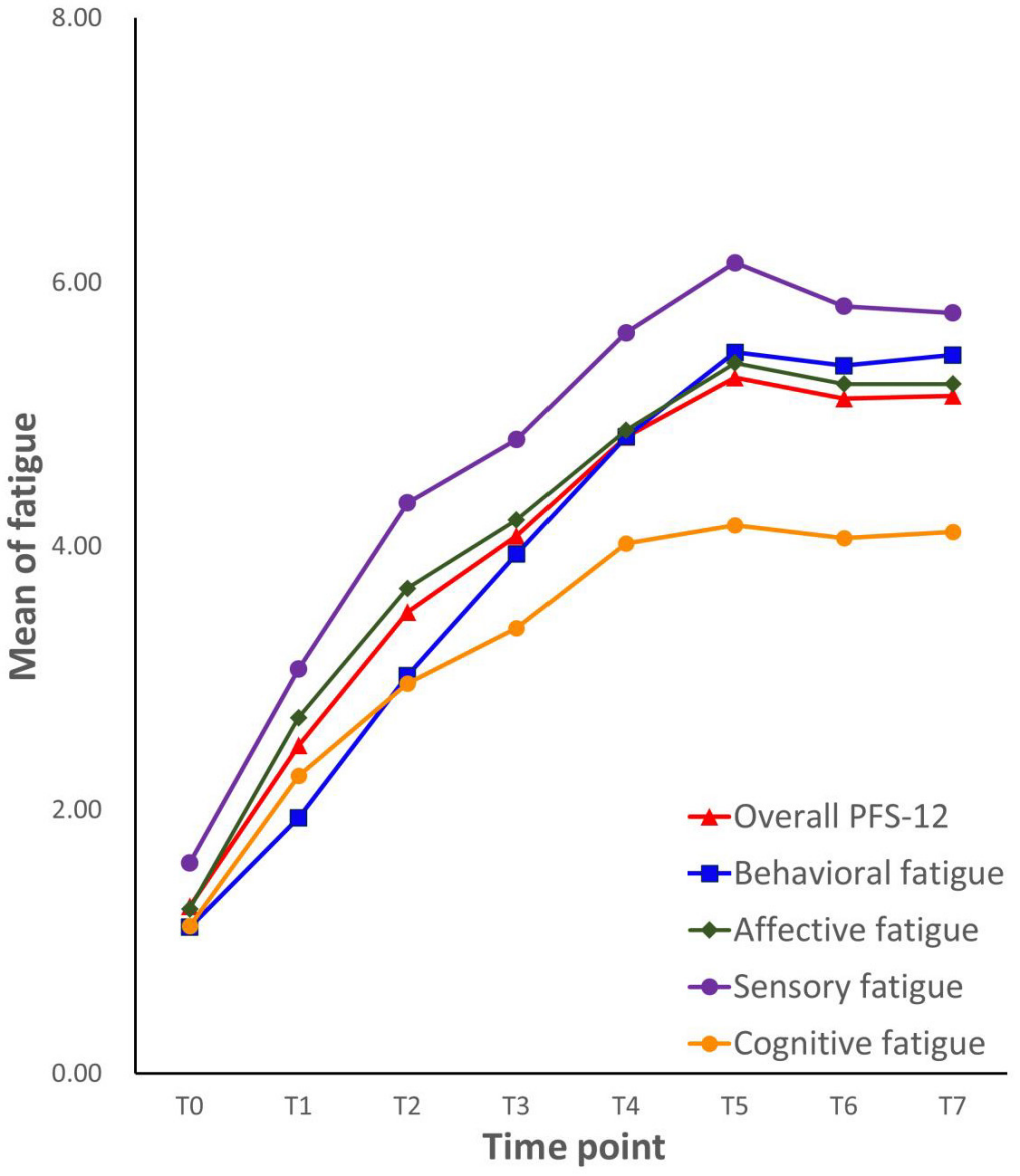
The trajectory of the PFS‐12.

The trajectory of the PFS‐12 showed a rising trend of a single peak (Figure 1 and Table 2) that was significantly affected over time. The mean overall fatigue at the initial point (T0) was 1.27 ± 1.82, increasing weekly, reaching the peak on T5 (5.28 ± 1.54), and declining slightly on the sixth and seventh weeks.

**TABLE 2:** The trajectories of overall PFS‐12 and four subscales, and nutrition status, body composition from T0 to T7.

Variables	T0 (*n* = 105)	T1 (*n* = 103)	T2 (*n* = 100)	T3 (*n* = 98)	T4 (*n* = 98)	T5 (*n* = 98)	T6 (*n* = 98)	T7 (*n* = 98)	*p* Value
Overall PFS‐12	1.27 ± 1.82	2.49 ± 1.72	3.50 ± 1.71	4.08 ± 1.73	4.83 ± 1.49	5.28 ± 1.54	5.12 ± 1.53	5.14 ± 1.80	<0.001
Behavioral fatigue	1.11 ± 1.86	1.94 ± 1.80	3.02 ± 1.86	3.94 ± 1.90	4.83 ± 1.71	5.47 ± 1.70	5.37 ± 1.76	5.45 ± 2.00	<0.001
Affective fatigue	1.25 ± 2.09	2.70 ± 1.97	3.68 ± 1.92	4.20 ± 1.84	4.88 ± 1.69	5.39 ± 1.83	5.23 ± 1.63	5.23 ± 2.01	<0.001
Sensory fatigue	1.60 ± 2.20	3.07 ± 2.15	4.33 ± 2.03	4.81 ± 2.10	5.62 ± 1.76	6.15 ± 1.57	5.82 ± 1.74	5.77 ± 1.91	<0.001
Cognitive fatigue	1.12 ± 1.78	2.26 ± 1.81	2.96 ± 1.75	3.38 ± 1.68	4.02 ± 1.59	4.16 ± 1.75	4.06 ± 1.72	4.11 ± 2.00	<0.001
PG‐SGA	2.97 ± 2.08	6.91 ± 4.03	11.16 ± 3.93	13.83 ± 5.06	16.50 ± 4.63	17.72 ± 4.08	17.93 ± 4.48	17.73 ± 5.00	<0.001
Body composition									
Weight	66.51 ± 10.87	66.70 ± 10.79	65.36 ± 10.76	64.80 ± 10.57	64.12 ± 10.50	63.24 ± 10.36	62.70 ± 10.30	62.25 ± 10.18	0.014
BMI	23.62 ± 3.30	23.70 ± 3.33	23.22 ± 3.35	23.03 ± 3.29	22.76 ± 3.24	22.47 ± 3.23	22.29 ± 3.21	22.13 ± 3.19	0.002
Body fat rate	25.77 ± 6.07	25.77 ± 6.02	25.05 ± 6.17	24.75 ± 6.08	24.33 ± 6.02	23.79 ± 6.14	23.52 ± 6.11	23.25 ± 6.23	0.015
Lean body weight	49.12 ± 7.16	49.24 ± 7.23	48.70 ± 7.22	48.50 ± 7.24	48.29 ± 7.21	47.94 ± 7.15	47.49 ± 7.39	47.50 ± 7.06	0.540

Abbreviations: BMI, body mass index; PFS‐12, the Piper Fatigue Scale‐12; PG‐SGA, The Scored Patient‐Generated Subjective Global Assessment.

The four PFS‐12 subscales tended to develop in the same direction over time (Table 2), whereas the development speeds differed. The sensory fatigue score was the highest from T0 to T7. The behavioral fatigue score was the lowest at T0, quickly increased from T1 to T5, and was higher than affective and cognitive/mood fatigue. And cognitive/mood fatigue was the third‐highest at T0, with the slowest ascending rate of the three dimensions and the lowest score at T7.

Following treatment, the PG‐SGA development curve was nearly parallel to the PFS‐12 and increased weekly, indicating that the nutritional status of the patients deteriorated. The peak appeared on T6, then fell slightly as the PFS‐12.

Table 2 depicts the trajectories of weight, body mass index, body fat rate, and lean body weight in patients who ascended mildly from T0 to T1 but descended weekly after T1.

### 3.3 The effect of demographic, clinical, baseline lifestyle, psychiatric, and social support characteristics on the trajectories of fatigue

Patients who were married, with higher hospital anxiety and depression, experienced severe total fatigue and each subscale (Table 3). Apart from behavioral fatigue, aging and lower resilience were distinguished from aggravating all the fatigue. Aside from sensory fatigue, patients with a drinking history reported reduced fatigue. Patients with three cycles of induced chemotherapy reported more behavioral and cognitive fatigue. Conversely, patients who experienced ≥2 CCRT cycles and had no smoking history reported less affective and sensory fatigue. Moreover, patients with the III tumor stage exhibited extreme behavioral fatigue, and those with ≥2.15 Gy fractionated doses presented less sensory fatigue. Physical activity at a high level at baseline was revealed to be a protective factor against cognitive fatigue.

**TABLE 3:** The effect of demographic and clinical characteristics, baseline psychiatric and social factors of participants on trajectories of overall PFS‐12 and subscales (univariate regression analysis).

Variable	Statistics (*N*,%)	PFS_T (β, 95%CI)	PFS_B (β, 95%CI)	PFS_E (β, 95%CI)	PFS_S (β, 95%CI)	PFS_C (β, 95%CI)
Sex						
Male	648 (77.14%)	0	0	0	0	0
Female	192 (22.86%)	0.23 (−0.12, 0.59)	0.22 (−0.18, 0.63)	0.20 (−0.19, 0.58)	0.32 (−0.09, 0.72)	0.19 (−0.15, 0.53)
Age	47.30 ± 11.58	0.02 (0.01, 0.03) **	0.01 (−0.00, 0.03)	0.02 (0.01, 0.03) **	0.02 (0.01, 0.04) ***	0.01 (0.00, 0.03) *
Settlement						
Country	312 (37.14%)	0	0	0	0	0
Town	264 (31.43%)	−0.02 (−0.39, 0.35)	−0.00 (−0.42, 0.41)	0.10 (−0.29, 0.50)	−0.18 (−0.59, 0.24)	−0.01 (−0.36, 0.34)
City	264 (31.43%)	−0.32 (−0.68, 0.04)	−0.33 (−0.73, 0.08)	−0.26 (−0.65, 0.13)	−0.41 (−0.83, −0.00)*	−0.25 (−0.59, 0.09)
Education						
Junior high and below	456 (54.29%)	0	0	0	0	0
Senior high and above	384 (45.71%)	0.04 (−0.26, 0.34)	0.03 (−0.31, 0.37)	0.05 (−0.27, 0.38)	−0.03 (−0.37, 0.31)	0.09 (−0.20, 0.37)
Marital status						
Never or Divorced	136 (16.19%)	0	0	0	0	0
Married	704 (83.81%)	1.11 (0.72, 1.50) ***	1.12 (0.68, 1.56) ***	1.12 (0.70, 1.54) ***	1.29 (0.85, 1.74) ***	0.87 (0.50, 1.24) ***
Family incoming						
≤5000 Y/month	328 (39.05%)	0	0	0	0	0
>5000 Y/month	512 (60.95%)	0.02 (−0.28, 0.33)	0.26 (−0.09, 0.60)	−0.01 (−0.34, 0.32)	−0.29 (−0.64, 0.06)	0.13 (−0.15, 0.42)
Smoking						
No	400 (47.62%)	0	0	0	0	0
Yes or ever	440 (52.38%)	0.29 (−0.01, 0.59)	0.25 (−0.09, 0.59)	0.35 (0.03, 0.67) *	0.35 (0.01, 0.69)*	0.22 (−0.06, 0.51)
Drinking						
No	488 (58.10%)	0	0	0	0	0
Yes or ever	352 (41.90%)	−0.36 (−0.66, −0.05) *	−0.47 (−0.82, −0.13) **	−0.40 (−0.72, −0.07) *	−0.25 (−0.60, 0.09)	−0.29 (−0.58, −0.00) *
UICC/AJCC stage						
II and below	128 (15.24%)	0	0	0	0	0
III and above	712 (84.76%)	0.23 (−0.18, 0.64)	0.51 (0.06, 0.97)*	0.16 (−0.28, 0.61)	−0.04 (−0.50, 0.43)	0.29 (−0.10, 0.67)
Induced chemotherapy cycle						
≤2 times	248 (29.52%)	0	0	0	0	0
≥3 times	592 (70.48%)	0.27 (−0.06, 0.59)	0.44 (0.07, 0.80)*	0.23 (−0.13, 0.58)	−0.02 (−0.39, 0.35)	0.43 (0.12, 0.74) **
CCRT cycle						
≤1 times	344 (40.95%)	0	0	0	0	0
≥2 times	496 (59.05%)	−0.28 (−0.59, 0.03)	0.06 (−0.29, 0.41)	−0.42 (−0.75, −0.09)*	−0.84 (−1.18, −0.49)***	0.08 (−0.21, 0.37)
Single radiation dose						
﹤2.15 Gy	560 (67.31%)	0	0	0	0	0
≥ 2.15 Gy	272 (32.69%)	−0.20 (−0.52, 0.12)	−0.10 (−0.46, 0.26)	−0.28 (−0.62, 0.06)	−0.47 (−0.83, −0.11)*	0.06 (−0.24, 0.36)
Total radiation dose						
﹤70 Gy	488 (58.65%)	0	0	0	0	0
≥70 Gy	344 (41.35%)	0.03 (−0.28, 0.33)	0.05 (−0.29, 0.39)	−0.05 (−0.38, 0.28)	−0.13 (−0.48, 0.22)	0.25 (−0.04, 0.54)
Resilience Scale	60.89 ± 19.95	−0.01 (−0.02, −0.00) **	−0.01 (−0.02, 0.00)	−0.01 (−0.02, −0.00)**	−0.02 (−0.03, −0.01) ***	−0.01 (−0.02, −0.00) **
Social Support Rating Scale	41.62 ± 7.26	−0.01 (−0.03, 0.01)	−0.01 (−0.03, 0.02)	−0.00 (−0.02, 0.02)	−0.00 (−0.02, 0.02)	−0.02 (−0.04, 0.00)
Hospital Anxiety and Depression Scale	12.64 ± 6.12	0.11 (0.09, 0.13) ^***^	0.08 (0.05, 0.11) ^***^	0.12 (0.09, 0.14) ^***^	0.13 (0.11, 0.16) ^***^	0.11 (0.09, 0.13) ^***^
Physical activity intensity						
Low	80 (9.62%)	0	0	0	0	0
Medium	248 (29.81%)	0.19 (−0.41, 0.79)	0.19 (−0.48, 0.87)	0.09 (−0.55, 0.74)	0.52 (−0.15, 1.20)	−0.07 (−0.63, 0.50)
High	504 (60.58%)	−0.23 (−0.80, 0.34)	−0.01 (−0.65, 0.62)	−0.20 (−0.82, 0.41)	−0.12 (−0.76, 0.52)	−0.58 (−1.11, −0.05)*

Abbreviations: CCRT, concurrent chemoradiotherapy; PFS_T, the average score of total items of Piper Fatigue Scale‐12; PFS_B, the average score of behavior subscale of Piper Fatigue Scale‐12; PFS_A, the average score of affective subscale of Piper Fatigue Scale‐12; PFS_S, the average score of sensory subscale of Piper Fatigue Scale‐12; PFS_C, the average score of cognitive/mood subscale of Piper Fatigue Scale‐12.

***p < 0.001; **p < 0.01; *p < 0.05.

Table 3 summarized education, family income, total radiation dose, and baseline social support not identified as effective on fatigue in patients during radiotherapy.

### 3.4 The longitudinal association between fatigue and nutrition status, body composition one week ahead

In general additive mixed models (Table 4), the PG‐SGA 1 week ahead was found to have a positive association with fatigue except for cognitive fatigue in the crude model. Only overall and behavior fatigue remained statistically significant with the PG‐SGA following adjustment in models I and II. The effect values varied from 0.0 to 0.05. There was no longitudinal association between fatigue and body composition 1 week ahead. Tables S1–S5 lists the confounders adjusted in each model.

**TABLE 4:** Multivariate analysis of nutrition, body composition a week ago on PFS‐12, and each subscale over time.

	GAMM [β (95%CI) *p*]			MSM [β (95%CI) *p*]
	Crude Model	Model I	Model II	Model I
Overall PFS‐12				
PG‐SGA	**0.03 (0.01, 0.06) 0.011**	**0.02 (0.00, 0.05) 0.044**	0.02 (−0.00, 0.05) 0.063	**0.15 (0.13, 0.17) <0.001**
Weight	−0.00 (−0.02, 0.02) 0.989	0.01 (−0.01, 0.03) 0.500	0.01 (−0.01, 0.03) 0.500	**−0.02 (−0.03, −0.00) 0.014**
BMI^§^	0.02 (−0.06, 0.09) 0.615	0.02 (−0.04, 0.08) 0.466	0.02 (−0.04, 0.08) 0.466	−0.04 (−0.08, −0.00) 0.058
Body fat rate	0.03 (−0.01, 0.07) 0.154	0.02 (−0.02, 0.05) 0.350	0.02 (−0.02, 0.05) 0.332	0.00 (−0.02, 0.03) 0.802
Lean body weight	−0.00 (−0.03, 0.03) 0.995	0.03 (−0.01, 0.06) 0.178	0.03 (−0.01, 0.06) 0.178	**−0.03 (−0.05, −0.01) 0.011**
Behavioral fatigue				
PG‐SGA	**0.05 (0.02, 0.08)<0.001**	**0.04 (0.02,0.07) 0.002**	**0.04 (0.02,0.07) 0.002**	**0.20 (0.18, 0.22) <0.001**
Weight	0.00 (−0.02, 0.03) 0.836	0.01 (−0.02, 0.03) 0.561	0.01 (−0.02, 0.03) 0.561	**−0.02 (−0.03, −0.00) 0.026**
BMI	0.01 (−0.07, 0.10) 0.725	0.01 (−0.06, 0.08) 0.706	0.01 (−0.06, 0.08) 0.706	**−0.05 (−0.10, 0.01) 0.029**
Body fat rate	0.03 (−0.01, 0.07) 0.175	0.02 (−0.03, 0.06) 0.454	0.02 (−0.03, 0.06) 0.454	−0.00 (−0.03, 0.02) 0.738
Lean body weight	0.01 (−0.03, 0.04) 0.729	0.04 (−0.01, 0.08) 0.128	0.04 (−0.01, 0.08) 0.128	−0.02 (−0.05, 0.00) 0.051
Affective fatigue				
PG‐SGA	**0.04 (0.01, 0.06) 0.018**	0.03 (−0.00, 0.06) 0.062	0.03 (−0.00, 0.06) 0.072	**0.15 (0.13, 0.17) <0.001**
Weight	0.01 (−0.02, 0.03) 0.695	0.01 (−0.01, 0.04) 0.271	0.01 (−0.01, 0.04) 0.271	−0.01 (−0.02, 0.00) 0.188
BMI	0.04 (−0.05, 0.12) 0.393	0.04 (−0.02, 0.11) 0.218	0.04 (−0.03, 0.11) 0.228	−0.02 (−0.07, 0.02) 0.376
Body fat rate	0.03 (−0.01, 0.08) 0.144	0.03 (−0.01, 0.07) 0.194	0.03 (−0.02, 0.07) 0.240	0.01 (−0.01, 0.03) 0.409
Lean body weight	0.01 (−0.03, 0.04) 0.778	0.03 (−0.01, 0.08) 0.117	0.03 (−0.01, 0.08) 0.117	−0.02 (−0.04, 0.00) 0.108
Sensory fatigue				
PG‐SGA	**0.04 (0.01, 0.07) 0.016**	0.02 (0.01, 0.05) 0.109	0.03 (−0.00, 0.06) 0.057	**0.16 (0.14, 0.18) <0.001**
Weight	0.00 (−0.03, 0.03) 0.928	0.01 (−0.01, 0.03) 0.525	0.01 (−0.01, 0.03) 0.525	**−0.02 (−0.03, −0.00) 0.024**
BMI	0.03 (−0.06, 0.11) 0.551	0.01 (−0.05, 0.08) 0.659	0.01 (−0.05, 0.08) 0.659	−0.04 (−0.08, 0.01) 0.144
Body fat rate	0.03 (−0.01, 0.08) 0.141	0.01 (−0.03, 0.05) 0.530	0.01 (−0.02, 0.05) 0.481	0.01 (−0.01, 0.03) 0.401
Lean body weight	−0.01 (−0.04, 0.03) 0.745	0.02 (−0.02, 0.06) 0.291	0.02 (−0.02, 0.06) 0.291	**−0.03 (−0.05, −0.01) 0.010**
Cognitive fatigue				
PG‐SGA	0.03 (−0.00, 0.06) 0.061	0.01 (−0.01, 0.04) 0.303	0.01 (−0.02, 0.04) 0.426	**0.11 (0.09, 0.13) <0.001**
Weight	−0.01 (−0.04, 0.01) 0.283	−0.00 (−0.03, 0.02) 0.699	−0.00 (−0.03, 0.02) 0.682	**−0.02 (−0.04, −0.01) <0.001**
BMI	−0.02 (−0.09, 0.06) 0.629	−0.00 (−0.06, 0.06) 0.999	−0.00 (−0.06, 0.06) 0.999	**−0.05 (−0.10, −0.01) 0.012**
Body fat rate	0.01 (−0.03, 0.05) 0.491	0.01 (−0.03, 0.05) 0.664	0.01 (−0.03, 0.05) 0.664	0.00 (−0.03, 0.02) 0.755
Lean body weight	−0.02 (−0.05, 0.03) 0.287	−0.00 (−0.04, 0.04) 0.865	−0.00 (−0.04, 0.04) 0.865	**−0.03 (−0.05, −0.01) <0.001**

*Note*: The models were fitted as Y = β_0_ + β_1_X + β_2_T + β_3_T^2^, time(T) was fitted as a continuous variable, and there was no interaction between variables (X) and time. Bold data indicated that *p*‐values were less than 0.05.

Abbreviations: BMI, body mass index; GAMM, the generalized additive mixed model; MSM, the marginal structural model; PFS‐12, the Piper Fatigue Scale‐12; PG‐SGA, the Scored Patient‐Generated Subjective Global Assessment.

However, in the MSMs (Table 4), when the HADS was controlled as a time‐dependent confounder, the PG‐SGA 1 week ahead showed positive associations with overall fatigue where all subscales and the effect values were increased (0.11 ~ 0.20). Furthermore, data revealed weight, BMI, and lean body weight 1 week ahead were statistically negative with fatigue, with the effect sizes ranging from −0.05 to −0.02.

## 4 DISCUSSION

In this longitudinal research, 3 GAMMs and 1 MSM were conducted to investigate the association of trajectories on CRF and nutrition status 1 week ahead in people with NPC during radiotherapy with/without CCRT. Based on our knowledge, this was the first study to consider the time window between nutrition status and multi‐dimension CRF in the above subjects.

The fatigue developed weekly in patients with NPC undergoing radiotherapy with/without chemotherapy. It approached the peak (the most severe) in the fifth week after treatment, similar to another longitudinal study.^3^ Most participants completed two concurrent chemotherapy cycles by the end of the fourth week. The incidence of clusters of symptoms peaked during the fifth week. Other studies in which fatigue was measured every 2 weeks (at the second, fourth, and sixth weeks)^18^, ^37^ did not observe a single peak. In another study, 73.10% of patients received more than three cycles of concurrent chemotherapy, and at 6 weeks of treatment, fatigue continued to increase.^2^ Chemotherapy and radiotherapy have been identified as independent factors contributing to fatigue. As treatment proceeds, cumulative and synergistic adverse reactions exacerbate fatigue.^38^, ^39^, ^40^ Therefore, 1 week following chemotherapy should be crucial for monitoring fatigue.

Further investigation was conducted on the variance of four subscales, which progressed in the same direction as overall fatigue, with sensory fatigue consistently scoring the highest. The behavioral fatigue set in the quickest, scoring lowest at T0 and swiftly rising to the second at T5. The findings were consistent with another study in patients with breast cancer.^18^, ^41^ The plausible explanation was that heaviness, bushiness, and numbness were the most common self‐reported symptoms that patients described as fatigue during treatment. And the subjective feeling that fatigue caused was stronger than the impact on activities of daily living. The reports indicated that it needs to be considered to promote physical function and positive feelings while developing the intervention strategies for CRF, for example, designing group activities or cooperative games.^42^, ^43^

Following treatment, the PG‐SGA development curve was parallel to the PFS‐12 and increased weekly, showing a deterioration in the nutritional status of the patients. The peak appeared on T6 before dipping a little. All the body composition indexes rose slightly at T1 and fell from the second week (T2). A similar result has been reported in the study by Ding.^44^ The trajectory charts of the above indexes hinted that there might be a time window issue with the changes in nutrition status and fatigue in patients with NPC during radiotherapy.

In the study, demographic factors that were examined to be associated with the trajectory of fatigue included marriage and age. Married patients reported increased fatigue in this study, which was not found in other research. The primary reason was that 12/17 patients in the singles group were under 20. Aging was distinguished from aggravating all the fatigue apart from behavioral fatigue, which is consistent with other studies.^4^

Fatigue was found to be influenced by various treatment and disease‐related factors, including tumor stage, fractionated dose, and concurrent and induced chemotherapy cycles. Patients with ≥III tumor stage experienced severe behavioral fatigue, similar to the other studies.^4^ Patients who experienced ≥3 cycles of induced chemotherapy before radiation reported higher behavioral and cognitive fatigue. However, patients who experienced ≥2.15 Gy fractionated dose and ≥2 CCRT cycles complained of less fatigue. The findings appeared paradoxical but logical, and there is an independent dose‐effect relationship between chemotherapy and radiotherapy on fatigue. Moreover, fatigue is one of the manifestations of the patient's tolerance to CCRT. The therapeutic regimen would be altered for those who showed severe fatigue, according to the studies of Pan^39^ and Chen.^4^

There were interesting findings on lifestyles in this study. Patients with a drinking history reported less fatigue, aside from sensory fatigue, as documented in previous studies.^45^, ^46^ Our explanation was alcohol could play an anti‐anxiety role by regulating the expression of the Hif3a gene in the amygdala,^47^ thus alleviating fatigue. In contrast, smoking history was identified as a risk factor for affective and sensory fatigue. There is evidence that the muscle of smokers are weaker and less fatigue‐resistant than those of nonsmokers.^48^ Patients with high‐intensity physical activity at baseline reported less cognitive fatigue due to improved physical function and endurance to fatigue.^49^, ^50^, ^51^ The results indicated that lifestyles may play an important role in the development of fatigue and should be considered in further intervention studies.

In psychosocial factors, anxiety and depression showed a positive correlation with fatigue, verified by other studies.^52^, ^53^, ^54^ Resilience was distinguished as a protective factor for all fatigue apart from behavioral fatigue, which is consistent with another study.^55^

The CRF and nutritional status/body composition are constantly changing in different stages of cancer patients.^3^ However, previous longitudinal studies focus on the relationship between baseline nutritional status/body composition and fatigue, ignoring the changes in nutritional status/body composition during treatment.^13^, ^56^, ^57^ This longitudinal research conducted three GAMMs, and only PG‐SGA was detected to have a statistically positive association with future‐week fatigue. However, the effects were weak but stable, varying from 0.02 to 0.04. The confounders were selected by the rule of “change‐in‐estimate”(CIE), in which confounders are selected based on the stability of the exposure effect estimator.^36^ As a comprehensive nutritional evaluation tool, the evaluation contents of PG‐SGA include the recent changes in patients' body weight, dietary intake, gastrointestinal symptoms, and physical activity,^24^ which is more predictive for fatigue than individual body composition indexes.

During cancer treatment, patients' emotions fluctuate over time, which has been demonstrated to be strongly correlated with fatigue.^6^, ^58^ So, HADS should be considered a time‐dependent confounder. MSMs were first adopted to explore the association between fatigue and nutritional status based on the Inverse Probability of Treatment Weighting.^59^ Intriguingly, the effect values of the positive relationships between the PG‐SGA increased dramatically to 0.12 ~ 0.20. Meanwhile, 1 week before, weight, BMI, and lean body weight showed statistically negative associations with fatigue, although the independent effects were minor. The results showed that the association between nutritional status/body composition, and fatigue could be covered if time‐dependent confounders were not controlled.

Cancer‐related fatigue is a subjective experience, and there is a lack of valid objective indicators to assess it. If only assessed by a self‐reported scale, it could induce unavoidable measurement bias. This study is an initial exploration of objective indicators of fatigue; future studies should probe more indicators that accurately reflect the degree of fatigue in cancer patients.

This study has some limitations. First, body composition indexes were achieved by BIA, which is convenient but has lower accuracy than a CT scan; Second, as an observational study, other confounders were considered; Third, nutrition status was assessed by body composition and PG‐SGA, other plasma/serum biomarkers were not included, which should be considered in the further studies.

## 5 CONCLUSION

The observational longitudinal study provided new evidence for CRF management in patients with NPC during treatment. First, the multidimensional fatigue in adults with NPC undergoing radiotherapy is dynamic, with the apex occurring during the fifth week, a critical time point that should be monitored more closely in future CRF management practice. The most severe form of fatigue is found to be sensory fatigue, whereas behavioral fatigue develops the fastest. Second, The PG‐SGA could predict the trend in fatigue 1 week before, when depression and anxiety were controlled as time‐dependent confounders in MSM. Body composition has a minor predictive value. Future research may explore the relationship between CRF and the loss of nutrition and body composition during treatment.

## AUTHOR CONTRIBUTIONS

**Jihong Song:** Formal analysis (equal); methodology (equal); writing – original draft (lead). **Xinru Yang:** Data curation (equal); investigation (equal); writing – review and editing (equal). **Jieling Wu:** Data curation (equal); investigation (equal); writing – review and editing (equal). **Zilan Wu:** Data curation (equal); investigation (equal); writing – review and editing (equal). **Litao Zhuo:** Data curation (equal); investigation (equal); writing – review and editing (equal). **Jinsheng Hong:** Conceptualization (equal); methodology (equal); writing – review and editing (equal). **Li Su:** Resources (equal); writing – review and editing (equal). **Wenlong Lyu:** Resources (equal); writing – review and editing (equal). **Jinru Ye:** Supervision (equal); writing – review and editing (equal). **Yan Fang:** Supervision (equal); writing – review and editing (equal). **Zhiying Zhan:** Formal analysis (equal); writing – review and editing (equal). **Hairong Zhang:** Formal analysis (equal); writing – review and editing (equal). **Xiaomei Li:** Conceptualization (equal); writing – review and editing (equal).

## FUNDING INFORMATION

This study was supported by grants from the Natural Science Foundation of Fujian Province, China [grant numbers 2023 J01699] and the CIFST – Abbott Foundation of Food Nutrition and Safety [grant numbers 2020–12].

## CONFLICT OF INTEREST STATEMENT

The authors have no conflicts of interest to disclose.

## ETHICS STATEMENT

The study protocol was approved by the Institutional Review Board (IRB) of Fujian Medical University (FMU2021[114]). Each participant provided informed content before each survey.

## Supporting information


Appendix S1.

## Data Availability

The data that support the findings of this study are available from the corresponding author upon reasonable request.
