# Supplementary material for: Could nutrition status predict fatigue one week before in patients with nasopharynx cancer undergoing radiotherapy?
Source: Cancer Med. 2024 Apr 25;13(8):e7191. doi: 10.1002/cam4.7191 (PMC11043677; doi:10.1002/cam4.7191)
Supplement: Supplementary file 1 — Appendix S1. [file CAM4-13-e7191-s001.docx]

103 participants finished the assessment at Tl

105 participants were recruited and finished the primary investigation at T0

2 quit radiotherapy for old age

1 went back to the local hospital for treatment

2 withdrew for personal reasons

100 participants finished the assessment at T2

98 participants finished all the 8 time-point assessments at T7

2 withdrew for personal reasons

**Supplement 1 Flow diagram of participants in the study.**

**Supplement 2 The adjusted covariates in generalize additive mixed models of Overall Fatigue and nutrition, body composition one-week ahead.**

|  | **PG-SGA** | | **Weight** | | **BMI** | | **Body fat rate** | | **Lean body weight** | |
| --- | --- | --- | --- | --- | --- | --- | --- | --- | --- | --- |
|  | **Model I** | **Model II** | **Model I** | **Model II** | **Model I** | **Model II** | **Model I** | **Model II** | **Model I** | **Model II** |
| **Sex** |  | √ | √ | √ | √ | √ | √ | √ | √ | √ |
| **Age** |  | √ | √ | √ | √ | √ | √ | √ | √ | √ |
| **Settlement** |  | √ | √ | √ | √ | √ |  | √ | √ | √ |
| **Education** |  |  | √ | √ | √ | √ | √ | √ | √ | √ |
| **Family incoming** |  | √ | √ | √ | √ | √ | √ | √ | √ | √ |
| **Marital status** |  | √ | √ | √ | √ | √ | √ | √ | √ | √ |
| **Smoking** |  | √ | √ | √ | √ | √ | √ | √ | √ | √ |
| **Drinking** |  | √ | √ | √ | √ | √ | √ | √ | √ | √ |
| **Tumor stage** |  | √ | √ | √ | √ | √ |  | √ | √ | √ |
| **Induced chemotherapy cycles** |  | √ | √ | √ | √ | √ | √ | √ | √ | √ |
| **CCRT cycle** |  | √ | √ | √ | √ | √ | √ | √ | √ | √ |
| **Total radiation dose^†^** |  |  | √ | √ | √ | √ | √ | √ | √ | √ |
| **Resilience Scale** |  | √ | √ | √ | √ | √ | √ | √ | √ | √ |
| **Social Support Rating Scale** |  |  | √ | √ | √ | √ | √ | √ | √ | √ |
| **Physical activity** |  | √ | √ | √ | √ | √ | √ | √ | √ | √ |
| **Hospital Anxiety and Depression Scale** | √ | √ | √ | √ | √ | √ | √ | √ | √ | √ |

^†^Single radiation dose was deleted due to collinearity diagnosis with total radiation dose.

**Supplement3 The adjusted covariates in generalize additive mixed models of Behavior Fatigue and nutrition, body composition one-week ahead.**

|  | **PG-SGA** | | **Weight** | | **BMI** | | **Body fat rate** | | **Lean body weight** | |
| --- | --- | --- | --- | --- | --- | --- | --- | --- | --- | --- |
|  | **Model I** | **Model II** | **Model I** | **Model II** | **Model I** | **Model II** | **Model I** | **Model II** | **Model I** | **Model II** |
| **Sex** |  |  | √ | √ | √ | √ | √ | √ | √ | √ |
| **Age** |  | √ | √ | √ | √ | √ | √ | √ | √ | √ |
| **Settlement** |  | √ | √ | √ | √ | √ | √ | √ | √ | √ |
| **Education** |  |  | √ | √ | √ | √ | √ | √ |  |  |
| **Family incoming** |  | √ | √ | √ | √ | √ | √ | √ | √ | √ |
| **Marital status** |  | √ | √ | √ | √ | √ | √ | √ | √ | √ |
| **Smoking** |  | √ | √ | √ | √ | √ | √ | √ | √ | √ |
| **Drinking** |  | √ | √ | √ | √ | √ | √ | √ | √ | √ |
| **Tumor stage** |  | √ | √ | √ | √ | √ | √ | √ | √ | √ |
| **Induced chemotherapy cycles** |  | √ | √ | √ | √ | √ | √ | √ | √ | √ |
| **CCRT cycle** |  |  | √ | √ | √ | √ |  |  |  |  |
| **Total radiation dose^†^** |  |  | √ | √ | √ | √ | √ | √ | √ | √ |
| **Resilience Scale** |  | √ | √ | √ | √ | √ | √ | √ | √ | √ |
| **Social Support Rating Scale** |  |  | √ | √ | √ | √ | √ | √ | √ | √ |
| **Physical activity** |  |  | √ | √ | √ | √ | √ | √ | √ | √ |
| **Hospital Anxiety and Depression Scale** | √ | √ | √ | √ | √ | √ | √ | √ | √ | √ |

^†^Single radiation dose was deleted due to collinearity diagnosis with total radiation dose.

**Supplement 4 The adjusted covariates in generalize additive mixed models of Affective Fatigue and nutrition, body composition one-week ahead**

|  | **PG-SGA** | | **Weight** | | **BMI** | | **Body fat rate** | | **Lean body weight** | |
| --- | --- | --- | --- | --- | --- | --- | --- | --- | --- | --- |
|  | **Model I** | **Model II** | **Model I** | **Model II** | **Model I** | **Model II** | **Model I** | **Model II** | **Model I** | **Model II** |
| **Sex** |  |  | √ | √ | √ | √ | √ | √ | √ | √ |
| **Age** |  | √ | √ | √ | √ | √ | √ | √ | √ | √ |
| **Settlement** |  | √ | √ | √ | √ | √ |  | √ | √ | √ |
| **Education** |  |  |  |  | √ | √ |  |  |  |  |
| **Family incoming** |  |  |  |  | √ | √ | √ | √ | √ | √ |
| **Marital status** |  | √ | √ | √ | √ | √ | √ | √ | √ | √ |
| **Smoking** |  | √ | √ | √ | √ | √ | √ | √ | √ | √ |
| **Drinking** |  | √ | √ | √ | √ | √ | √ | √ | √ | √ |
| **Tumor stage** |  |  | √ | √ | √ | √ |  |  | √ | √ |
| **Induced chemotherapy cycles** |  | √ | √ | √ | √ | √ |  | √ | √ | √ |
| **CCRT cycle** |  | √ | √ | √ |  | √ | √ | √ | √ | √ |
| **Total radiation dose^†^** |  |  | √ | √ | √ | √ | √ | √ | √ | √ |
| **Resilience Scale** |  | √ | √ | √ | √ | √ | √ | √ | √ | √ |
| **Social Support Rating Scale** |  |  | √ | √ | √ | √ | √ | √ | √ | √ |
| **Physical activity** |  |  | √ | √ | √ | √ | √ | √ | √ | √ |
| **Hospital Anxiety and Depression Scale** | √ | √ | √ | √ | √ | √ | √ | √ | √ | √ |

^†^Single radiation dose was deleted due to collinearity diagnosis with total radiation dose.

**Supplement 5 The adjusted covariates in generalize additive mixed models of Sensory Fatigue and nutrition, body composition one-week ahead**

|  | **PG-SGA** | | **Weight** | | **BMI** | | **Body fat rate** | | **Lean body weight** | |
| --- | --- | --- | --- | --- | --- | --- | --- | --- | --- | --- |
|  | **Model I** | **Model II** | **Model I** | **Model II** | **Model I** | **Model II** | **Model I** | **Model II** | **Model I** | **Model II** |
| **Sex** |  | √ | √ | √ | √ | √ | √ | √ | √ | √ |
| **Age** |  | √ | √ | √ | √ | √ | √ | √ | √ | √ |
| **Settlement** |  | √ | √ | √ | √ | √ | √ | √ | √ | √ |
| **Education** |  |  | √ | √ | √ | √ |  | √ | √ | √ |
| **Family incoming** |  |  | √ | √ | √ | √ | √ | √ | √ | √ |
| **Marital status** | √ | √ | √ | √ | √ | √ | √ | √ | √ | √ |
| **Smoking** |  | √ | √ | √ | √ | √ | √ | √ | √ | √ |
| **Drinking** |  |  | √ | √ | √ | √ | √ | √ | √ | √ |
| **Tumor stage** |  |  | √ | √ | √ | √ |  |  | √ | √ |
| **Induced chemotherapy cycles** |  |  | √ | √ | √ | √ | √ | √ | √ | √ |
| **CCRT cycle** |  | √ | √ | √ | √ | √ | √ | √ | √ | √ |
| **Total radiation dose^†^** |  |  | √ | √ | √ | √ | √ | √ | √ | √ |
| **Resilience Scale** | √ | √ | √ | √ | √ | √ | √ | √ | √ | √ |
| **Social Support Rating Scale** |  |  | √ | √ | √ | √ | √ | √ | √ | √ |
| **Physical activity** |  |  | √ | √ | √ | √ | √ | √ | √ | √ |
| **Hospital Anxiety and Depression Scale** | √ | √ | √ | √ | √ | √ | √ | √ | √ | √ |

^†^Single radiation dose was deleted due to collinearity diagnosis with total radiation dose.

**Supplement 6 The adjusted covariates in generalize additive mixed models of Cognitive Fatigue and nutrition, body composition one-week ahead.**

|  | **PG-SGA** | | **Weight** | | **BMI** | | **Body fat rate** | | **Lean body weight** | |
| --- | --- | --- | --- | --- | --- | --- | --- | --- | --- | --- |
|  | **Model I** | **Model II** | **Model I** | **Model II** | **Model I** | **Model II** | **Model I** | **Model II** | **Model I** | **Model II** |
| **Sex** |  |  | √ | √ | √ | √ | √ | √ | √ | √ |
| **Age** |  | √ | √ | √ | √ | √ | √ | √ | √ | √ |
| **Settlement** |  | √ |  |  |  | √ | √ | √ |  | √ |
| **Education** |  |  |  |  | √ | √ | √ | √ |  |  |
| **Family incoming** |  |  |  |  | √ | √ | √ | √ | √ | √ |
| **Marital status** |  | √ | √ | √ | √ | √ | √ | √ | √ | √ |
| **Smoking** |  | √ | √ | √ | √ | √ | √ | √ | √ | √ |
| **Drinking** |  | √ | √ | √ | √ | √ | √ | √ | √ | √ |
| **Tumor stage** |  | √ |  | √ | √ | √ | √ | √ |  | √ |
| **Induced chemotherapy cycles** |  | √ | √ | √ | √ | √ | √ | √ | √ | √ |
| **CCRT cycle** |  |  |  |  |  |  |  |  |  |  |
| **Total radiation dose^†^** |  | √ | √ | √ | √ | √ | √ | √ | √ | √ |
| **Resilience Scale** |  | √ | √ | √ | √ | √ | √ | √ | √ | √ |
| **Social Support Rating Scale** |  | √ | √ | √ | √ | √ | √ | √ | √ | √ |
| **Physical activity** |  | √ | √ | √ | √ | √ | √ | √ | √ | √ |
| **Hospital Anxiety and Depression Scale** | √ | √ | √ | √ | √ | √ | √ | √ | √ | √ |

^†^Single radiation dose was deleted due to collinearity diagnosis with total radiation dose.
